# Comparison of clinical outcomes of three internal fixation techniques in the treatment of olecranon fracture: a retrospective clinical study

**DOI:** 10.1186/s12891-022-05482-8

**Published:** 2022-06-01

**Authors:** Hongfei Qi, Zhong Li, Yao Lu, Teng Ma, Shuai Ji, Bing Du, Ming Li, Qiang Huang, Kun Zhang, Yanling Yang

**Affiliations:** 1 Department of Orthopaedics and Trauma, Hong Hui Hospital, Xi’an Jiaotong University College of Medicine, No. 555, East Youyi Road, Xi’an, 710000 Shaanxi China; 2 Medical College of Yan’an University, No. 30, Guanghua Road, Baota District, Yan’an, 716000 Shaanxi China

**Keywords:** Olecranon fracture, Double-plate, Tension band wiring, LCP, Elbow fracture

## Abstract

**Objective:**

The application of double plating in olecranon fractures is becoming increasingly widespread. There is no research comparing this technique with traditional tension band wiring (TBW) and the single plate technique. The purpose of this study was to compare the efficacy of three fixation techniques in olecranon fractures.

**Materials and methods:**

From March 2016 to May 2020, we collected the clinical data of 95 patients with olecranon fractures who underwent surgical treatment. Thirty-five patients received TBW surgery (TBW Group), 32 patients received a 3.5 mm locking compression plate (LCP, 3.5 mm LCP Group), and 28 patients received double mini-locking plate treatment (DP Group). The operation time, fracture union time, time of return to work, range of motion (ROM), soft tissue stimulation to remove internal fixation, and patient-related functional results (the Weseley score, Mayo Elbow Performance Score [MEPS], and Disabilities of Arm, Shoulder and Hand Score [DASH]) were recorded. The clinical results and complications of the three internal fixation techniques were compared.

**Results:**

The average follow-up time was 15.011.82 months (12–18 months). All patients’ fractures healed by first intention. There were no statistically significant differences in the operation time, fracture union time, ROM, Weseley score, MEPS or DASH scores of the three groups of patients. The postoperative return time for patients in the TBW group was 10.002.15 weeks, the 3.5 mm LCP group was 9.561.93 weeks, and the DP group was 8.432.38 weeks (*P* = 0.014); 12 patients in the TBW group required removal of plant due to soft tissue stimulation, the 3.5 mm LCP group had 8 cases, and the DP group had 2 cases (*P* = 0.038).

**Conclusion:**

The postoperative clinical results and elbow joint function of patients with olecranon fractures fixed by tension band wiring, 3.5 mm LCP and double mini-locking plate are similar, which indicates that double-plate technology can be used as an alternative to the two groups of traditional techniques. In addition, double-plate technology also helps patients return to work earlier and has a lower incidence of soft tissue stimulation.

## Background

The olecranon is an important part of the elbow joint. It works with the coronoid process of the ulna, distal humerus, radial head and ligament structure of the elbow joint to maintain the stability of the elbow joint. Olecranon fracture is a relatively common type of fracture, accounting for approximately 10% of fractures around the elbow joint and 1% of total fractures [1, 2]. Olecranon fractures are usually caused by falls or traffic accidents [3]. Due to the tension of the triceps, most fractures may not be suitable for conservative treatment [4, 5]. Simple proximal olecranon fractures are usually treated with tension band wiring (TBW) [6]. TBW is a simple and low-cost technique [7]. Its disadvantages include the high number of symptomatic prominences of K-wires and the technique’s limited capability in more complex fractures [8, 9]. For complex and comminuted fractures, plates are usually selected for surgical treatment [10, 11]. The plate is placed on the dorsal side of the ulna to fix the fracture [12]. Its common complications include wound healing problems and the occurrence of soft tissue irritation, even after the initial healing, which requires removal of internal plates [13].

In 2010, Rochet et al. [14] proposed the double-plate technique to treat olecranon fractures. They described placing two one-third tubular plates on the medial and lateral sides of the ulna. The results of a biomechanical study by Hoelscher-Doht et al. [15] showed that double-plate fixation can provide high stability under high load conditions while reducing the stimulation of soft tissues. Soft tissue stimulation after olecranon fractures is the most common complication [16]. The double-plate technique may be an effective solution [17]. However, there is no research to compare the clinical efficacy of the three fixation techniques. Therefore, our study reviewed the clinical data and postoperative functional status of patients with olecranon fractures who underwent surgical treatment in our centre from March 2016 to May 2020 and compared the clinical efficacy of these three fixation techniques in the treatment of olecranon fractures.

### Data and methods

Inclusion criteria: 1. Diagnosed with olecranon fracture; 2. Age > 18 years; 3. Good joint function before elbow injury; 4. Complete follow-up data.

Exclusion criteria: 1. Patients with fractures of other parts of the elbow joint (distal humerus, radial head, coronoid process); 2. Patients with severe complications and inoperable; 3. Nondisplaced fracture fragments or fragment displacement < 2 mm; 4. Time from injury to operation > 2 weeks; 5. Open fractures.

From March 2016 to May 2020, Hong Hui Hospital, affiliated with Xi’an Jiaotong University, admitted a total of 143 patients with olecranon fractures, of which 31 patients had fractures of other parts of the elbow joint and 4 patients were excluded due to preoperative elbow joint dysfunction. Thirteen patients that failed to complete follow-up were excluded. According to the inclusion and exclusion criteria, a total of 95 patients were enrolled in this study, of which 35 patients were treated with TBW, 32 patients were treated with 3.5 mm locking compression plates (LCPs), and 28 patients were treated with double mini-locking plate (DP) technology. Their demographic characteristics and general information are shown in Table 1. This study was approved by the ethics committee of Xi’an Hong Hui Hospital, and all patients included in the study signed informed consent forms.

**Table 1:** Patient demographics and basic information

Variable	TBW Group (35)	3.5 mm LCP Group (***n*** = 32)	DP Group(***n*** = 28)	***P*** value
Patient characteristics				
Gender (female/male)	15/20	13/19	11/17	0.958^b^
Age (year)	47.66±13.09	43.91±13.85	46.21±10.39	0.419^a^
BMI	24.21±3.01	24.33±3.23	23.82±2.83	0.800^a^
Cause of Trauma				0.782^b^
Fall	25	25	20	
Traffic Accident	10	7	8	
ASA score				0.685^b^
I	22	20	16	
II	9	11	10	
III	4	1	2	
Mayo Classification				0.150^b^
Ia	0	0	0	
Ib	0	0	0	
IIa	21	19	13	
IIb	13	11	9	
IIIa	1	2	3	
IIIb	0	0	3	
Average length of stay (day)	7.40±1.87	7.25±1.92	7.36±1.66	0.943^a^

Intergroup comparisons performed using ANOVA or the Chi-square test (a ANOVA; b Chi-square test)

### Surgical treatment

#### DP group

**Fig. 1:**
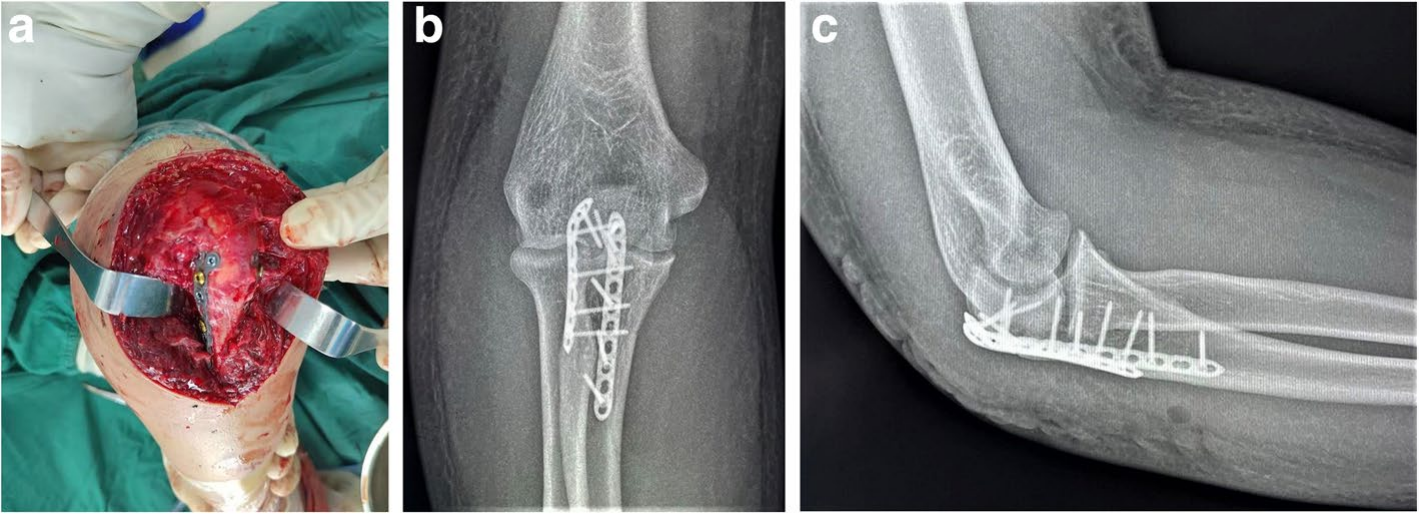
**a**: Place plates on the posterior medial and posterior lateral sides of the olecranon; **b-c**: anteroposterior and lateral X-ray film of the elbow joint

With the patient supine and the arm dropped across the chest, a posterior surgical approach was performed. Point forceps were used for reduction, and Kirschner wire was used for temporary fixation. After checking the flatness of the joint surface under fluoroscopy, a 2.7 mm system mini bone plate was used to shape along the bone surface on both sides of the olecranon of the ulna across the fracture line. The length of the bone plate can determine its span and screw fixation site according to the fracture shape (according to the fracture shape, different plate types can be selected for fixation, such as straight type, arc type, T type, Y type, etc.). Usually, cortical bone screws are driven into the holes on both sides of the fracture line to obtain close contact between the bone plate and the bone surface and realize compression between the broken ends of the fracture. The rest can be fixed with locking screws or cortical screws. Intraoperative examination should be conducted to ensure that elbow flexion, extension and rotation activities are unimpeded. X-ray fluoroscopy should be used to check the fracture reduction and fixation of bone plate screws, place drainage, and close the wound layer by layer (Fig. 1).

#### 3.5 mm LCP group

**Fig. 2:**
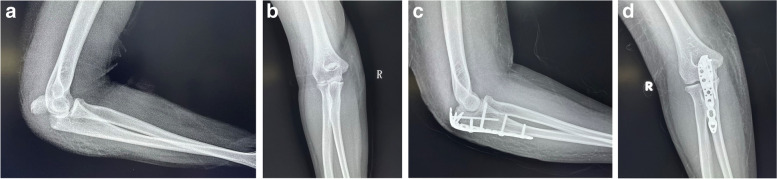
A 47-year-old male underwent surgery with 3.5 mm LCP. **a b**. Anteroposterior and lateral X-ray film of the elbow joint before operation. **c d**. Postoperative anteroposterior and lateral X-ray film of the elbow joint

The surgical position and surgical incision of the 3.5 mm LCP group were the same as those of the DP group. After the fracture was clearly exposed, the fracture was reduced under direct vision. Generally, the compressed fracture was reduced first, and the distal fracture end and humeral trochlea were used as the standard. The collapsed fracture in the joint was pried and reset to reset it to the distal end. The main fracture ends and matches the humerus trochlear surface. Finally, the elbow was extended to reset the proximal fracture of the triceps brachii tendon, and the fracture was temporarily fixed with Kirschner wire. Then, an olecranon 3.5 mm locking compression plate was placed on the proximal back of the ulna and fixed with screws (Fig. 2). After confirming satisfactory fracture reduction and fixation, the wound was closed layer by layer.

#### TBW group

**Fig. 3:**
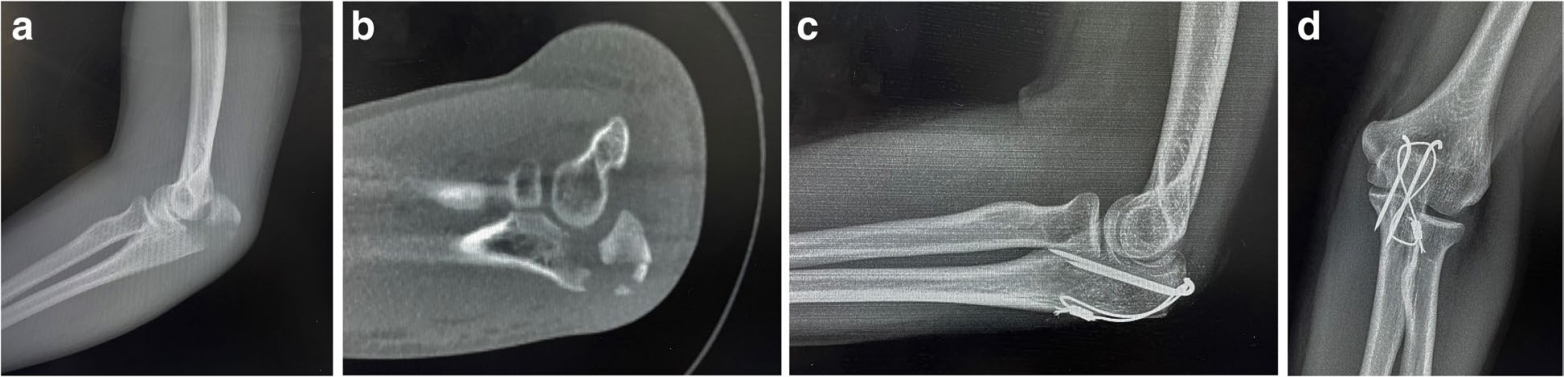
A 36-year-old male underwent surgery with TBW. **a** X-ray film of the lateral position of the elbow joint before the operation; **b** Plain CT scan of the elbow joint before the operation; **c d** Postoperative anteroposterior and lateral X-ray film of the elbow joint

The TBW group also used a midline posterior elbow incision and reduction forceps to reduce the fracture. Two parallel Kirschner wires are drilled through the fractured end to make them parallel to the long axis of the ulna and pass through both sides at the distal end of the fracture line approximately 2 mm. A bone hole with a diameter of 2 mm was drilled across the cortical bone, a 0.5 mm steel wire was inserted through the bone hole, the protruding Kirschner wire was crossed in a figure of eight, the steel wire was tightened and ligated, and the curved Kirschner wire was hammered into the bone (Fig. 3). After checking the fracture reduction and fixation satisfactorily, the wound was closed layer by layer.

### Postoperative rehabilitation

All patients recovered according to the standard rehabilitation plan. On the second day after the operation, the drainage tube was removed, antibiotics were applied for 24 hours (cefuroxime sodium is the routine antibiotic choice, and clindamycin is the choice for patients with allergies or contraindications), and the elbow joint extension brace was fixed on the first 3 days after the operation. Passive elbow movement was started from the 4th day, and only the elbow joint was passively moved within 2 weeks postoperatively. The number of activities was not too high, but each time the patient should try to reach the maximum range of elbow joint flexion and extension activities. The upper limbs were suspended at 90° elbow flexion. After 2 weeks, active functional exercises were started. After 4 weeks, the patient was allowed to do daily activity training without resisting resistance or gravity.

### Efficacy evaluation

In the first 3 months after surgery, all patients underwent outpatient review once a month and complete X-ray examinations. The standard of fracture union is that X-ray film shows that there is continuous callus passing through the fracture line, and the fracture line disappears completely; the clinical standard of fracture union is that the patient can live without elbow pain.

The situation of patients in each group requiring implant removal due to soft tissue stimulation was recorded. At the last follow-up, the maximum ROM and the function of the elbow joint were evaluated. The maximum ROM of the elbow joint is measured by placing the affected limb on a standard angle measurement table. Regarding the functional results of the elbow joint, we used the Weseley score, the Mayo Elbow Performance Score (MEPS) and DASH Scores for evaluation. The Weseley score is an objective score that evaluates the results of pain and loss of function, and the results are rated as fair, good, and excellent. The MEPS is evaluated in three aspects: pain, elbow joint stability and daily activities. The rating is excellent at 90–100 points, good at 75–89 points, good at 60–74 points, and poor at 0–59 points. The DASH score is a tool for scoring the function of upper extremity diseases [18]. It asks patients how difficult it is to perform physical activities. The evaluation results range from 100 points (serious difficulties) to 0 points.

### Statistical analysis

Data analysis was performed using SPSS version 13.0 (SPSS Inc., Chicago, IL, USA). Continuous variables were reported as the mean and standard deviation. One-way analysis of variance (ANOVA) was used to compare the differences among multiple groups. The Chi-square test was used for the analysis of categorical data. A *P* value < 0.05 indicated a statistically significant difference.

## Results

The demographic characteristics and general information of the 3 groups of patients were not significantly different, and they were comparable. The average follow-up time was 15.011.82 months. No patients had serious complications of deep infection or internal fixation failure. The average operation time was 96.7119.81 min in the TBW group, 104.9122.12 min in the 3.5 mm LCP group, and 107.0017.12 min in the DP group (*P* = 0.093). The fracture union time in the TBW group was 9.142.13 weeks, that in the 3.5 mm LCP group was 9.882.72 weeks, and that in the DP group was 9.462.08 weeks (*P* = 0.442). At the last follow-up, the elbow ROM of the TBW group was 117.2012.12°, that of the 3.5 mm LCP group was 120.3114.37°, and that of the DP group was 120.2111.29° (*P* = 0.527). The Wesley score showed excellent results in 19 cases in the TBW group. The 3.5 mm LCP group was excellent in 20 cases, and the DP group was excellent in 22 cases (*P* = 0.314). The MEPS showed excellent results in 19 cases in the TBW group. The 3.5 mm LCP group was excellent in 18 cases, and the DP group was excellent in 18 cases (*P* = 0.518). The DASH score of the TBW group was 18.1710.35, that of the 3.5 mm LCP group was 15.038.90, and that of the DP group was 12.757.80 (*P* = 0.067). The above indicators were not significantly different among the 3 groups of patients. However, the TBW group was 10.002.15 weeks, the 3.5 mm LCP group was 9.561.93 weeks, and the DP group was 8.432.38 weeks (*P* = 0.014). In the TBW group, 12 patients requested removal of the implant due to soft tissue stimulation, 8 patients requested removal in the 3.5 mm LCP group and 2 patients requested removal in the DP group (*P* = 0.038, Table 2).

**Table 2:** Comparison of clinical results of three internal fixation techniques

Variable	TBW Group (35)	SP Group (***n*** = 32)	DP Group (***n*** = 28)	***P*** value
Operation time (min)	96.71±19.81	104.91±22.12	107.00±17.12	0.093^a^
Union Times (week)	9.14±2.13	9.88±2.72	9.46±2.08	0.442^a^
Final ROM (°)	117.20±12.12	120.31±14.37	120.21±11.29	0.527^a^
Weseley				0.314^b^
excellent	19	20	22	
good	14	11	6	
fair	2	1	0	
MEPS				0.518^b^
excellent	19	18	18	
good	13	13	10	
fair	3	1	0	
DASH	18.17±10.35	15.03±8.90	12.75±7.80	0.067^a^
Time of return to Work (week)	10.00±2.15	9.56±1.93	8.43±2.38	0.014^a^
Implant removal by soft tissue irritation	12	8	2	0.038^b^
Follow up time (month)	14.80±1.91	15.38±1.66	14.86±1.88	0.381^a^

Intergroup comparisons were performed using ANOVA or the Chi-square test (a ANOVA; b Chi-square test)

**Fig. 4:**
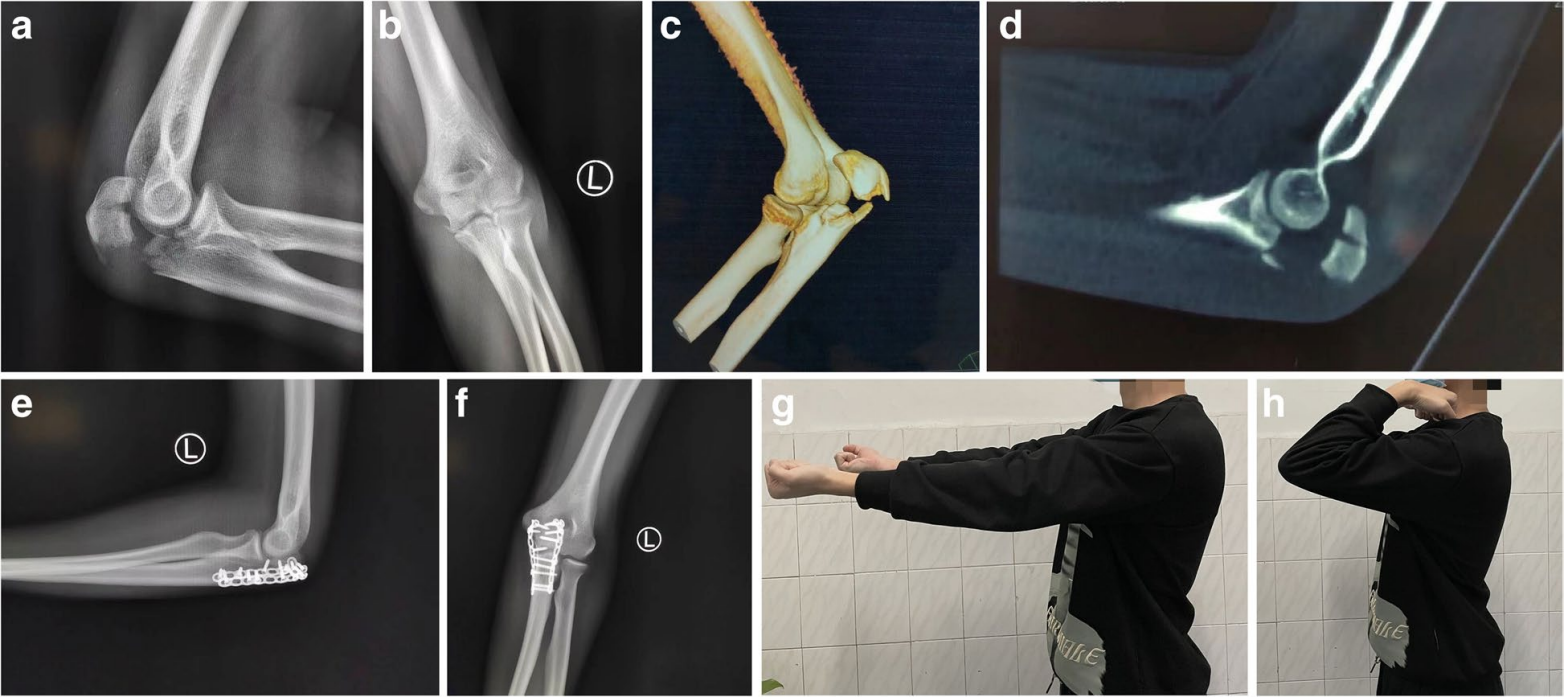
A 26-year-old man underwent surgery with double mini-locking plates. **a b c d** Anteroposterior and lateral X-ray film and three-dimensional CT of the elbow joint before the operation; **e f** postoperative anteroposterior and lateral X-ray film of the elbow joint; **g h** image of the patient’s elbow joint function after surgery

A typical case is shown in Fig. 4. A 26-year-old male patient received double mini-locking plate treatment. The patient recovered well after the operation and obtained the same elbow joint function as the healthy side.

## Discussion

The treatment of elbow fractures mainly depends on whether the joint is dislocated, the severity of the joint injury, and the stability of the elbow joint. The Mayo classification mainly classifies fracture displacement, comminution and joint stability and has guiding significance for clinical treatment [19]. The purpose of surgical treatment of olecranon fractures is to restore the anatomical position of the olecranon and perform early postoperative functional exercises. For comminuted fractures, the fracture fragments need to be fixed in a stable position to provide sufficient stability for fracture healing.

Common internal fixations for olecranon fractures are TBW [20], locking compression plates [11], one-third tubular plates [21] and reconstruction plates [22]. Among them, TBW is considered the gold standard for the treatment of simple olecranon fractures [23]. However, the ordinary Kirschner wire has a smooth design, no thread or tooth pattern, and a low holding force on the bone tunnel. The patient is prone to withdraw the needle during the rehabilitation process. Even skin ulceration has occurred, the end of the Kirschner wire was exposed, and the internal fixation had to be removed by a second operation [8, 24]. For complex, unstable, and comminuted fractures, TBW may not meet the requirements of fixation. In this case, plate fixation is usually selected. A randomized controlled study by Duckworth et al. [25] showed that plate fixation has achieved good clinical results and function after the treatment of olecranon fractures, but patients undergoing TBW surgery have a higher frequency of revision surgery. For patients undergoing plate fixation, due to the wide design of the plate, many patients can touch the plate and screws under the skin. In particular, the contours of the plate that appear when the elbow joint is flexed in thinner or young women may affect appearance, and skin irritation can also cause pain and discomfort in the patient [13]. To avoid such complications, Ries et al. [26] applied the double-plate technique in the surgical treatment of olecranon fractures. Two low-profile plates are placed on the posterior inner and posterior outer sides of the olecranon, rather than on the most prominent dorsal side, which may reduce the irritation of soft tissues to a certain extent. In addition, the method of placing plates on both sides to fix the strength may be more reliable, which was confirmed in a biomechanics study [15, 17].

A retrospective clinical study reported by Ellwein et al. [27] compared the clinical outcomes and postoperative complications of 3.5 mm LCP and low-profile double plates in the treatment of olecranon fractures. The results showed that the final ROM of patients undergoing 3.5 mm LCP surgery was 130 ± 21°, patient satisfaction was 91%, and the incidence of implant removal due to soft tissue irritation was 36%. The ROM of patients who underwent low-profile double-plate surgery was 127 ± 15°, patient satisfaction was 69%, and the incidence of implant removal due to soft tissue irritation was 24%. Although there was no significant difference between the two groups of patients in the above three indicators, the incidence of implant removal by soft tissue irritation was 12% lower in the low-profile double-plate group than in the 3.5 mm LCP group, which may indicate that the low-profile double-plate is less irritating to the skin and soft tissues.

Our research results show that patients in the DP group returned to work earlier after surgery and had fewer cases of implant removal due to soft tissue stimulation. Although it is more expensive, DP may have unique advantages for elderly patients with osteoporosis and complex fractures. From a mechanical point of view, bilateral fixation can obtain an arch effect similar to the parallel fixation of distal humeral fractures. The use of bone plate fixation on both sides of the olecranon can play a better role in clamping and fixing the broken end of the fracture. At the same time, due to the use of a 2.7 mm microscrew, it is easier to hold and fix the smaller proximal olecranon bone block than the 3.5 mm system. In the comparison of operation time, there was no significant difference between the three internal fixation methods. This may be because the surgical approach is the same, and there is not much difference in the exposure and fixation of the fracture. Therefore, the seemingly complicated dual-plate technique does not significantly increase the operation time. Based on our clinical practice, we believe that the advantages of the double miniature plate are as follows. First, the miniature plate is small in size and can be buried under the lateral elbow muscle and flexor carpi ulna at the posterior edge of the ulna to make it lower than the ridge on the olecranon side of the ulna, reducing the occurrence of skin irritation to the soft tissue of the plate. Second, the double plate technology can have a side blocking effect on the fracture fragments, reduce the fretting of the fracture end and make the fracture union smooth. Finally, double miniature plate fixation has little effect on the movement of the olecranon in the olecranon fossa, which is conducive to the early functional exercise of the patient after the operation and avoids the occurrence of elbow stiffness.

The main limitation of this study is that it is a retrospective study with a small sample size, which may cause a certain bias to the results of the study. Second, although all operations were performed by experienced orthopaedic specialists, the bias caused by different surgeons cannot be completely avoided. Third, the follow-up time of this study was short, and a longer follow-up might yield more interesting results.

## Conclusion

The clinical results and elbow joint function of patients with olecranon fractures fixed by TBW, 3.5 mm LCP and the double mini-locking plate technique are basically similar. Double mini-locking plate fixation is beneficial for patients to return to work earlier after surgery, and it also has fewer cases of removal of implant due to soft tissue stimulation. The double-plate technique can be used as an option for surgical treatment of olecranon fractures. Of course, our conclusion needs to be confirmed by a larger sample of randomized controlled clinical studies.

## Data Availability

The data that supported the findings of this study are available on request from the corresponding author. The data are not publicly available due to privacy or ethical restrictions.
